# Application of Response Surface Methodology and Desirability Function in the Optimization of Adsorptive Remediation of Arsenic from Acid Mine Drainage Using Magnetic Nanocomposite: Equilibrium Studies and Application to Real Samples

**DOI:** 10.3390/molecules24091792

**Published:** 2019-05-09

**Authors:** Aphiwe Siyasanga Gugushe, Azile Nqombolo, Philiswa N. Nomngongo

**Affiliations:** 1 Department of Chemical Sciences (Formerly known as Department of Applied Chemistry, University of Johannesburg, Doornfontein Campus), University of Johannesburg, Doornfontein Campus, P.O. Box 17011, Johannesburg 2028, South Africa; gugushe75@gmail.com (A.S.G.); azilenqombolo@gmail.com (A.N.); 2 DST/Mintek Nanotechnology Innovation Centre, University of Johannesburg, Doornfontein 2028, South Africa; 3 DST/NRF SARChI Chair: Nanotechnology for Water, University of Johannesburg, Doornfontein 2028, South Africa

**Keywords:** arsenic, central composite design, acid mine drainage, regeneration, MWCNT-Fe_3_O_4_@Zeo nanocomposite, two and three-parameter isotherm

## Abstract

A magnetic multi-walled carbon nanotube/zeolite nanocomposite was applied for the adsorption and removal of arsenic ions in simulated and real acid mine drainage samples. The adsorption mechanism was investigated using two-parameter (Langmuir, Freundlich, Temkin) and three-parameter (Redlich–Peterson, and Sips) isotherm models. This was done in order to determine the characteristic parameters of the adsorptive removal process. The results showed that the removal process was described by both mono- and multilayer adsorptions. Adsorption studies demonstrated that a multi-walled carbon nanotube/zeolite nanocomposite could efficiently remove arsenic in simulated samples within 35 min. Based on the Langmuir isotherm, the adsorption capacity for arsenic was found to be 28 mg g^−1^. The nanocomposite was easily separated from the sample solution using an external magnet and the regeneration was achieved by washing the adsorbent with 0.05 mol L^−1^ hydrochloric acid solution. Moreover, the nanoadsorbent was reusable for at least 10 cycles of adsorption-desorption with no significant decrease in the adsorption capacity. The nanoadsorbent was also used for the arsenic removal from acid mine drainage. Overall, the adsorbent displayed excellent reusability and stability; thus, they are promising nanoadsorbents for the removal of arsenic from acid mine drainage.

## 1. Introduction

Excessive discharge of arsenic into the environment has become a major issue due to its toxicity to fauna and flora [1]. Inorganic arsenic in aqueous solution at certain pH and redox conditions is found predominantly in the third oxidation state and the penta-oxidation state namely, arsenious acid (AsO_3_^3−^) and arsenate oxyanions (AsO_4_^3−^) respectively [2]. Arsenic also exists in organic form, e.g., arsenocholine, arsenobetaine and dimethylarsinous acid, however, these are not as harmful as the inorganic forms [3,4]. Mining, smelting, petroleum industry, combustion of fossil fuel in power plants and the use of arsenical pesticides and herbicides have contributed a lot towards the arsenic contamination in surface water and groundwater [5,6]. Excess exposure to arsenic can lead to damaging of the skin, lung, kidney, stomach, blood cell, brain and is also believed to be carcinogenic [1,4,7]. This has resulted in the WHO (World Health Organisation), European Commission (EC) and United State Environmental Protection Agency (USEPA) making the maximum amount of arsenic allowed in drinking water to be 10 µg L^−1^ [1,4,6,8]. The Ministry of Science and Technology of Thailand has set the minimum contamination level (MCL) allowed the industries to emit to be 250 µg L^−1^ [4]. Therefore, arsenic as a contaminant needs to be removed from mining and industrial waste to prevent the intake by living organisms.

The removal of arsenic in water is generally achieved by the employment of methods such as coprecipitation [9], ion-exchange [10], electrocoagulation [11], oxidation [12], co-precipitation [13], membrane technology [5], Donnan dialysis [14], reverse osmosis [6] and adsorption [1,4,8]. These methods are effective in removing arsenic from water, however, besides adsorption, they require expensive equipment, materials. In addition, they produce large quantities of sludge that needs to be disposed of safely and appropriately. Also, they are not easy to operate, and they may be able to effectively detect heavy metals in trace amounts [4,15]. The adsorption process has attracted much attention due to excellent properties such as environmental friendliness, ease of operation and cost-effectiveness, high efficiency [16,17,18]. The performance of the adsorption mainly depends on the adsorbent used, hence there has been many research efforts made to find efficient and cost-effective adsorbents [19]. These economic yet promising adsorbents include zeolites, chitin, chitosan, and agricultural wastes such as maize leaf, banana pith, peanut husk, pumpkin waste, rice hull and saw dust [15]. Recently, nanomaterials have received extensive attention in the field of metal/metalloid adsorption and environmental remediation [20]. This is because they have a high surface area-to-volume ratio that enables a much greater extraction capacity and efficiency as well as an easily modifiable surface functionality to improve the selectivity [21,22]. These nanoadsorbents include functionalized poly(glycidyl methacrylate) [23], functionalized nickel-iron oxide nanoparticles [24], starch-bridged magnetite nanoparticles [10] and amine functionalized silica particles with a magnetic core [25].

Batch adsorption methodology is usually optimized by means of monitoring one factor at time (OFAT) [26,27]. However, the limitation of this optimization approach is that it requires significant large number of experiments to performed for each factor, thus leading increasing consumption of reagent and materials [26,27]. Moreover, the OFAT approach is time consuming the interaction effects among the experimental parameters are not considered [26,27]. This can be overcome by employing multivariate approach such as response surface methodology (RSM). The RSM is defined as a collection of mathematical and statistical tools that can be used for the analysis of the effects of several experimental parameters (independent variables) [26,28,29,30,31]. RSM allows the evaluation of interaction of factors that may influence adsorption of the analytes of interest and is able to provide optimal conditions for desired responses [26,28,29,30,31]. Several researchers have reported the application RSM for the optimization of batch adsorption procedures [27,28,29,30,31].

In this study, magnetic multi-walled carbon nanotubes/zeolite (MWCNT-Fe_3_O_4_@Zeo) nanocomposite was applied for the removal of arsenic from simulated and acid mine drainage samples. A multivariate approach was used to investigate the possible influential factors, such as contact time, pH and mass of adsorbent. The experimental design was done using a response surface methodology called the central composite design (CCD). The performance of MWCNT-Fe_3_O_4_@Zeo nanocomposite was examined by equilibrium isotherm study. The influence of various competitive anions was studied the regeneration was performed in order to authenticate the potential reusability and stability. The novelty of this study lies on the application of a magnetic multi-walled carbon nanotube/zeolite nanocomposite for the first time as an adsorbent for the remediation of acid mine drainage.

## 2. Results and Discussion

### 2.1. Brunauer, Emmett, and Teller Surface Area Measurement

The surface area of MWCNTs, magnetic MWCNTs, natural zeolite and MWCNT-Fe_3_O_4_@Zeo nanocomposite were found to be 210, 137, 37.8 and 178 m^2^ g^−1^, respectively. The pore sizes of MWCNTs, magnetic MWCNTs, natural zeolite and MWCNT-Fe_3_O_4_@Zeo were 18.3, 14.9, 12.7 and 16.7 nm, respectively. Whereas the pore volumes were found to be 0.813, 0.532, 0.237 and 0.753 cm^3^ g^−1^ for MWCNTs, magnetic MWCNTs, natural zeolite and MWCNT-Fe_3_O_4_@Zeo, respectively. It was observed that the incorporation of Fe_3_O_4_ reduced the surface area of MWCNTs. This might be due to the formation of Fe_3_O_4_ on the pore structures of MWCNTs. However, the incorporation of zeolite on the surface of magnetic MWCNTs provide more adsorption sites, thus increasing the surface properties of the nanocomposite.

### 2.2. Optimization Strategy

Optimization is used to find the best suitable conditions for the interaction of the adsorbent and the sample (including analyte). Firstly, one must be aware of the possible variables that might affect the adsorption efficiency. In this study, a multivariate optimization was utilized to investigate the effect of the mass of adsorbent (MA), extraction time (ET) and sample pH on the adsorption efficiency. Specifically, the full factorial design (FFD) and central composite design (CCD) based on response surface methodology were used to determine the optimum conditions.

#### 2.2.1. A 2^3^ Full Factorial Design

**Figure 1:**
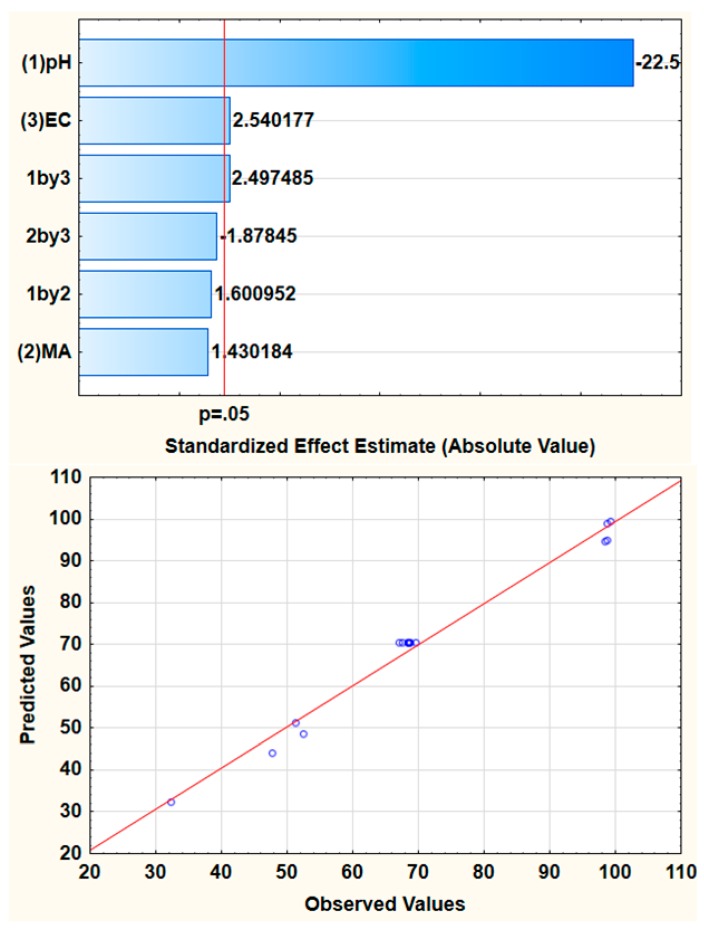
Pareto chart of standardized effects and relationship between actual and predicted %RE for the removal of arsenic.

The FFD was used to screen the most influential parameters for the adsorptive removal of arsenic in AMD. Table 1 presents the detailed FFD matrix of actual variables and the experimentally obtained percentage removal efficiency of arsenic (%RE, analytical response). The experiments were carried out randomly and the central points were repeated eight times in order to obtain better accuracy of the analytical response. As seen in Table 1, a maximum % removal of As reached 98.9% when using the as-prepared MWCNT-Fe_3_O_4_@Zeo nanocomposite. The experimental data in Table 1 were analyzed using analysis of variance (ANOVA) represented by Pareto chart (Figure 1). It is apparent from Figure 1 that sample pH and extraction time (ET), as well as their interactive effects, were statistically significant at 95% confidence level. This implied that the two independent variables were influential to the % RE because the bars that represent them pass the confidence limit line. Therefore, the two variables require further optimization. The predicted and observed responses (Figure 1) maintained a good relationship because they were equitably scattered around the straight line plot which is between the two sets of values. The predicted and adjusted R^2^ values of 0.9833 and 0.9722 were obtained and they were found to be in agreement, suggesting that the model was significant.

#### 2.2.2. Response Surface Methodology based on Central Composite Design

**Figure 2:**
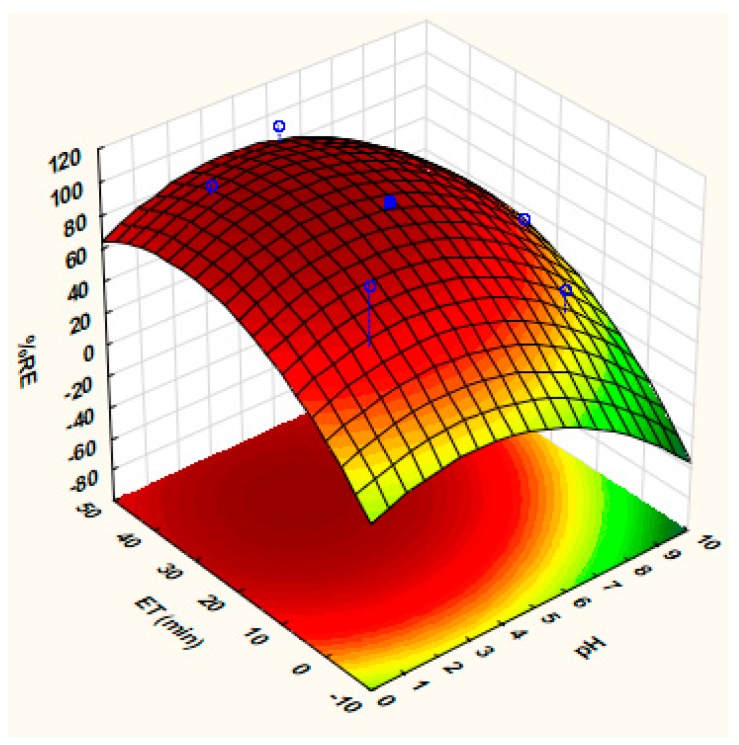
3-D Response surface plot for the %removal efficiency of As onto MWCNT-Fe_3_O_4_@Zeo nanocomposite: Effect of sample pH and extraction time (ET) when the mass of adsorbent fixed at 125 mg.

The effect of extraction time and sample on the percentage removal efficiency of arsenic onto the as-prepared nanocomposite was further investigated through response surface methodology based on CCD and the design matrix with corresponding responses are presented in Table 2. Figure 2 shows the response surface plot of the effect of pH and extraction time on the adsorption of arsenic onto MWCNT-Fe_3_O_4_@Zeo nanocomposite. The plot shows that arsenic analytical response (% removal efficiency) increased with decreasing sample pH. This phenomenon was explained based on the point of zero charge of the nanoadsorbent (PZC = 7.3) determined as described in Section 3.4. This implied that the MWCNT-Fe_3_O_4_@Zeo nanocomposite surface had a positive charge when solution pH is less than PZC, thus leading to increased arsenic removal efficiency. In addition, when the pH of is greater than PZC, the surface of the adsorbent becomes negatively charged thus resulting on decreased As removal. Furthermore, an increase in arsenic removal efficiency was observed with increasing extraction time (Figure 2). These finding suggested that that the diffusion of the analytes onto the pores of the adsorbent increased with time.

#### 2.2.3. Optimization of Adsorption Process Using the Desirability Function

**Figure 3:**
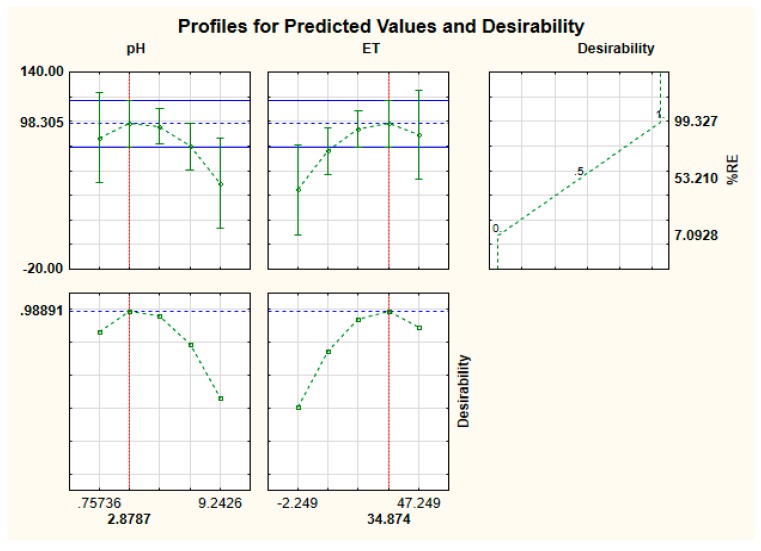
Desirability function for optimization of the adsorption process.

The desirability function was used as a numerical optimization tool in order to select desired values for each variable and the analytical response [30]. To achieve this, the two variables (ET and MA) were evaluated at a specific range of values, while the analytical response was aimed to attain a maximum [30,32]. Based on these conditions, the maximum arsenic % removal efficiency obtained was 98.3% (Figure 3) at sample pH of 2.9 and extraction of 35 min. Therefore, based on the FFD, RSM and desirability function, the optimum conditions were 2.9, 125 mg and 35 min for pH, MA and ET, respectively. In order to investigate the applicability of the optimum condition, the affirmative experiments were performed using 2.0 mg L^−1^. These results demonstrated that the removal efficiency of 99.8 ± 2.3% was attainable under these conditions. These results were in agreement with the predicted value of 98.3 which suggested the appropriateness and accuracy of the FFD and RSM models.

### 2.3. Adsorption Isotherms

The equilibrium data for the adsorption of arsenic on MWCNT-Fe_3_O_4_@Zeo was studied by fitting it in Langmuir, Freundlich, Temkin, Redlich-Peterson Hill and Sips linearized equations.

#### 2.3.1. Two Parameter Isotherms

The Langmuir isotherm assumes that the adsorbent surface is homogeneous(monolayer) and the energy of the adsorption sites is equal [33,34]. This means when an adsorbate occupies a site on the surface of the adsorbent, no further adsorption can take place on that site [33,34]. As seen in Table 3, the dimensionless constant factor (R_L_ = $\frac{1}{1+K_{L}C_{0}}$, where *K_L_* and *C*_0_ are Langmuir constant and initial concentration) values are in the range of 0 < R_L_ < 1 indicating that the adsorption of As on MWCNT-Fe_3_O_4_@Zeo is favourable [34,35]. The calculated Langmuir monolayer adsorption capacity of 27.8 mg/g. The Freundlich isotherm is based on multilayer adsorption where the energy of the adsorption sites increases exponentially [34,36]. It is said that the stronger sites are occupied first during adsorption [34]. When n values are between 1 and 10 they assume a favourable adsorption and in this work n = 3 thus the adsorption of arsenic on MWCNT-Fe_3_O_4_@Zeo was favorable [33,34,37]. The Temkin isotherm takes into consideration the indirect interactions between the adsorbent and the adsorbate on adsorption isotherms and because of these interactions it is said that the heat of adsorption layer decreases linearly rather than logarithmically [33,34,38].

#### 2.3.2. Three Parameter Isotherms

Redlich-Peterson isotherm has three unknown parameters namely, K_RP_, β and α_RP_ where maximization of the correlation coefficient was used in a linear plot to find the coefficients [36]. The exponent β is supposed to be between 0 and 1 and in this study, it was found to be 0.67 (Table 3) which is very close to unity. These observation implied that the adsorption data was described by Langmuir model rather than that of Freundlich isotherm [39]. The Sips model is ideal for describing heterogeneous surface adsorption surfaces adequately [40]. When the exponent n_s_ = 1 the adsorption followed the Langmuir isotherm whereas if n > 1 the experimental data fit the Freundlich isotherm more [41]. In this work n is approximately 1 and is thus a monolayer adsorption. Furthermore, the Hill isotherm model was applied to describe the adsorption of As. According to Hill isotherm model, depending on the value of n_H_, one can expect at least three possibilities; n_H_ > 1 means positive cooperativity in binding, n_H_ = 1 means hyperbolic binding or non-cooperative and n < 1 means negative cooperativity in the binding [34,42]. In this study, nH for adsorption of was <1 (Table 3), implying that the binding interaction between arsenic and the adsorbent was in the form of negative cooperativity [34,43]. In addition, the theoretical maximum adsorption capacity of Hill model was found to be 29.3 mg/g which is close to the experimental vale of 29.1 mg/g.

#### 2.3.3. Coefficients of Determination

The correlation coefficients found for the fit of the experimental data on the different isotherms were in this order, Langmuir > Hill > R-P > Sips > Freundlich > Temkin. Even though the Langmuir and Freundlich isotherms had correlation coefficients that are closely related. The adsorption isotherm mostly followed was the Freundlich as the adsorption was a multilayer adsorption proven by the β exponent of the R-P isotherm that is more less than 1 and reduces to the Freundlich model. The ns value of the Sips model was found to be more than unity and thus describes a heterogeneous adsorption. The maximum adsorption capacity from the Sips model was 28 mg/g.

The adsorption capacities of arsenic onto different sorbents described in the literature are summarised in Table 4. As seen in this table, the values of adsorption capacity in this study are better than those reported in the literature [44,45]. However, the adsorption capacity was lower than those reported elsewhere (Table 4). It worth mentioning that the adsorption capacity depends on the experimental conditions and surface characteristics of an adsorbent, this is why different uptake capacities for arsenic are observed.

### 2.4. Regeneration and Recycling of MWCNT-Fe_3_O_4_@Zeo Nanocomposite

**Figure 4:**
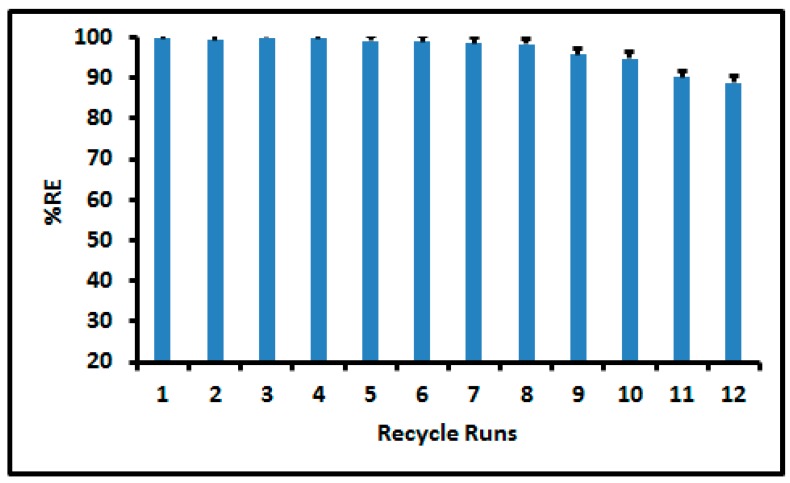
Percentage removal efficiency versus recycle runs for the regenerated MWCNT-Fe3O4@Zeo nanocomposite for the removal of As at optimal conditions performed at room temperature. Experimental conditions: 125 mg nanoadsorbent, 15 mL of 2.0 mg L^−1^ As solution, pH 2.8 and adsorption time 35.

In this study, dilute solution of HCl was used for regeneration of MWCNT-Fe_3_O_4_@Zeo nanoadsorbent and it was discovered that 0.5 mol L^−1^ HCl was sufficient enough to desorb As from the adsorbent. The stability of the nanoadsorbent (MWCNT-Fe_3_O_4_@Zeo) was assessed by checking the number of cycles with respect to the adsorption of 2.0 mg L^−1^ As using the same adsorbent for successive cycles. The adsorbent was separated by an external magnet and regenerated by 0.5 mol L^−1^ HCl. As seen from Figure 4, the nanoadsorbent could be reused for up to 10 adsorption/desorption cycles.

### 2.5. Application to Real Samples

The average initial arsenic concentrations in AMD samples analyzed are presented in Table 5 After passing the samples through nanoadsorbent the arsenic concentrations in the samples were below environmental quality standards for municipal and liquid industrial effluents (1000 µg L^−1^) recommended by National Environmental Quality Standards, Pakistan (NEQS, 2000). These results represented an average total percentage removal efficiency of 98.0%.

## 3. Materials and Methods

### 3.1. Instrumentations

Inductively coupled plasma optical emission spectroscopy (ICP-OES) was performed on a Thermo ICAP 6500 duo spectrometer (Thermo Scientific, England, UK), that has a charge injection device (CID) detector. The Branson 5800 ultrasonic Cleaner (Danbury, CT, USA) was used to assist the adsorption process. A pH meter with an electronic glass electrode (H1 9811–5, (HANNA Instruments, Smithfield, Rhode Island, USA) was used to adjust the pH of the solutions. Pore size distributions, Brunauer Emmett Teller apparatus (BET) surface areas and pore volumes were measured by nitrogen adsorption/desorption using Micromeritics, ASAP2020 (Micromeritics, Norcross, GA, USA).

### 3.2. Reagents and Solutions

All reagents used were of analytical grade unless stated otherwise and ultra-pure (type 1 with 18 MΩ cm^−1^ resistivity) deionized water from a Milli-Q water purification system (Millipore, Bedford, MA, USA) was used to prepare all solutions. Iron(III) chloride hexahydrate (FeCl_3_·6H_2_O) and iron(II) chloride tetrahydrate (FeCl_2_·4H_2_O), zeolite, carbon nanotubes used to synthesize the magnetic nanoadsorbent were purchased from Sigma-Aldrich (Fluka, St. Loius, MO, USA). The hydrochloric acid (HCl (37%), acetic acid and ultrapure nitric acid (69%) were purchased from Sigma-Aldrich (Fluka, St. Loius, MO, USA). Ammonia solution (25% *v*/*v*) used for syntheses and adjusting pH was purchased from Associated Chemical Enterprises, (Pty) Ltd. (Johannesburg, South Africa,). Fresh arsenic standard solutions in 1% (*v*/*v*) nitric acid (Sigma) and model solutions were prepared by accurately diluting appropriate volumes of a 1000 mg L^−1^ certified arsenic stock solution (Spectrascan TeknoLab, Drøbak, Norway).

### 3.3. Preparation of MWCNT-Fe_3_O_4_@Zeo Nanocomposite

The procedure used for the synthesis of the nanocomposite was carried out according to Javanbakht [49] method. Firstly, 0.5 g of zeolite and 0.5 g of MWCNTs were added to 60 mL of deionized (DI) water and was homogenized using an ultrasonic bath for 10 min. The FeCl_2_·4H_2_O and FeCl_3_·6H_2_O salts with a ratio of 2:1 were dissolved in ultrapure deionized water. The pH of the two solutions was adjusted to pH 2. The homogenized MWCNT and zeolite solution were then added to the FeCl_2_·4H_2_O and FeCl_3_·6H_2_O solution with continuous stirring under inert conditions at 70 °C. Then 30 mL of 5 M NaOH was added. After all the additions were made, stirring was continued for 60 min and the magnetic nanocomposite was separated using a magnetic field. The product was washed and dried at 70 °C for 10 h. From our previous studies, the characterization techniques confirmed the successful synthesis of the nanocomposite.

### 3.4. Determination of pH Point of Zero Charge (pHzpc) of MWCNT-Fe_3_O_4_@Zeo Nanocomposite

The pH_zpc_ of MWCNT-Fe_3_O_4_@Zeo nanocomposite was investigated by following the method described by [50]. To describe the procedure briefly, about 0.05 g the adsorbent was placed to 20 mL of approximately 0.1 M NaCl solutions. The solutions were adjusted to pH values between 2 and 12, using dilute solutions (1.0 mol L^−1^) of either HCl or NaOH. The solutions containing the adsorbent were agitated for 48 hours and the final pH values were then recorded. The change in pH (ΔpH) values were obtained by calculating the difference between the initial and final pH values. A plot of ΔpH vs. pH_f_ was obtained in order to obtain the pH_zpc_ which is defined as a pH value ΔpH of zero [50].

### 3.5. Multivariate Optimization of the Adsorption Process

Portions (15 mL) of 2.0 mg L^−1^ synthetic solutions of arsenic (pH 2.0–8.0) were placed in polypropylene sample bottles containing a specific amount of adsorbent (from 50–200 mg). The bottles were then sealed and put in a sonicator where they were subjected to sonic waves for 5–40 min so that the arsenic can be extracted from the sample solution to the nanoadsorbent. The liquid phase and the solid material were separated by external magnetic decantation. The samples were filtered into a pre-cleaned centrifuge tube and analyzed using the ICP-OES to find the residual concentration of the arsenic. The percentage removal efficiency (%RE) of the analyte was calculated using Equation (1)

(1) $$\%RE=\frac{C_{0}-C_{e}}{C_{0}}\times 100$$

where *C*_0_ (mg L^−1^) is the initial concentration and *C_e_* (mg L^−1^) is the concentration of arsenic after adsorption. The data obtained were processed using Statistica software (Version 13).

### 3.6. Adsorption Isotherms

An adsorption isotherm describes the amount of analyte that is in the supernatant compared to the amount that is in the adsorbent at equilibrium [36]. In this study, the adsorption of arsenic on MWCNT-Fe_3_O_4_@Zeo nanocomposite was studied under the obtained optimum conditions at room temperature. Samples at different concentrations (1–10 mg L^−1^) were prepared and each of them was adjusted to the optimum pH using dilute acetic acid and dilute ammonium hydroxide. A 15 mL aliquot of each of the samples was put in a sample bottle containing 125 mg of adsorbent and the bottles were sealed and placed into a sonicator. The samples were sonicated for 35 min. After separation of the aqueous phase from the sorbent using an external magnetic field, the liquid solution was analyzed using ICP-OES. Then, Equation (2) was used to determine the equilibrium adsorption capacity (*q_e_*, mg g^−1^):

(2) $$q_{e}=\frac{(C_{0}-C_{e})V}{m}$$

where *C*_o_ (mg/L) initial concentration of arsenic, *C_e_* is the equilibrium concentration of arsenic, *V* is the volume of the sample (L), and m(g) is the mass of the adsorbent used.

The equilibrium data was evaluated using five isotherm models, that is, Langmuir, Freundlich, Temkin, Sips and Redlich-Peterson (R-D) isotherms. These models were also used studied to determine the interaction between the sample solution containing the analyte of interest and the adsorbent.

• **Langmuir Isotherm Model**

Equation (3) was used to determine whether the experimental data fit the Langmuir Model which would mean the interaction between the adsorbent and the sample solution with the analyte is a monolayer (chemisorption) adsorption [35,51].

(3) $$\frac{C_{e}}{q_{e}}=\frac{1}{q_{\max}K_{L}}+\frac{C_{e}}{q_{\max}}$$

where *K_L_* is the Langmuir constant related to the energy of adsorption and *q*_max_ is the maximum adsorption capacity (mg/g).

• **Freundlich Isotherm Model**

The Freundlich isotherm is a combination of both monolayer and multilayer adsorptions. Equation (4) was used to determine how the equilibrium data fit this model [51,52].

(4) lnqe=lnKF+1nlnCe

where *K_F_* is Freundlich constants and n is the heterogeneity coefficient.

• **Temkin Isotherm Model**

The Temkin isotherm model is presented in Equation (5) it is based on the assumption that the adsorption energy decreases linearly with the surface coverage due to adsorbent–adsorbate interactions [51,52].

(5) $$q_{e}=B_{T}\ln K_{T}+B_{T}\ln C_{e},B_{T}=\frac{RT}{b_{T}}$$

where *b_T_* is the Temkin constant related to the heat of sorption (J/mol) and *K_T_* is the Temkin isotherm constant (L/g).

• **Redlich-Peterson (R-P) Isotherm Model**

The R-P isotherm is a combination of both the Langmuir isotherm and the Freundlich isotherm and is thus a three-parameter equation [33,53,54]. This equation may be used over a wide range of concentrations and its linear equation is presented by Equation (6) [53,54].

(6) $$\ln(K_{RP}\frac{C_{e}}{q_{e}}-1)=\beta \ln C_{e}+\ln\alpha_{RP}$$

where *K_RP_* (L g^−1^), *α_RP_* (L mg^−1^) and *β* are the Redlich–Peterson isotherm constants.

• **Sips Isotherm**

This isotherm proposes an empirical formula with three parameters, it is a hybrid of the Langmuir and Freundlich isotherm, it is more effective in describing adsorption on heterogeneous surfaces [40,41]. At different sorbate concentrations, it reduces to either the Freundlich isotherm or the Langmuir isotherm [40,41,53]. The Linear equation that represents the Sips equation is Equation (7) [53].

(7) $$\frac{1}{q_{e}}=\frac{1}{q_{\max}K_{s}}{\left(\frac{1}{C_{e}}\right)}^{\frac{1}{n}}+\frac{1}{q_{\max}}$$

where *K_S_* is Sips isotherm model constant (L g^−1^) and ‘1/n’ (which is numerically equal to ‘m’) is the Sips isotherm model exponent; “n” is obtained by using the trial and error method that gives the isotherm graph with correlation coefficient closest to 1 [53].

• **Hill Isotherm**

This model is normally used to describe the binding of analyte/adsorbate onto homogeneous adsorbent. In other words, Hill model signifies the general condition of Langmuir model [43,55]. The Hill isotherm is shown in Equation (8) [34,43,56]

(8) $$q_{e}=q_{H}\frac{C_{e}^{n_{H}}}{K_{D}+C_{e}^{n_{H}}}$$

where *n_H_* and *K_D_* are Hill isotherm constants and *q_H_* is maximum equilibrium adsorption capacity (mg/g).

## 4. Conclusions

This study demonstrated the effective application of a magnetic multi-walled carbon nanotube/zeolite (MWCNT-Fe_3_O_4_@Zeo) nanocomposite for the removal of arsenic from simulated and acid mine drainage. The effect of the most influential parameters, that is their individual and their interactions on the percentage removal efficiency of arsenic in real and model samples was measured. First order and second-order polynomial were fitted to the experimental data using full factorial and response surface methodology based on central composite design. Under optimized conditions, the maximum adsorption capacity of 20.4 mg g^−1^ for MWCNT-Fe_3_O_4_@Zeo nanocomposite and it was comparable to other studies reported elsewhere. The equilibrium adsorption data of MWCNT-Fe_3_O_4_@Zeo nanocomposite is described using the linear of Langmuir, Freundlich Temkin, Sips and Redlich–Peterson isotherm models. On the basis of β and n_s_ values, Redlich–Peterson and Sips isotherms suggested that the isotherms are approaching the Langmuir model, not the Freundlich isotherm. The MWCNT-Fe_3_O_4_@Zeo nanocomposite was applied for the removal of arsenic in real AMD samples and the results prove that the current adsorbent was effective in the adsorption of As in complex matrices. The MWCNT-Fe_3_O_4_@Zeo nanocomposite was easily regenerated by very dilute hydrochloric acid. The recycled nanocomposite could be reused 10 times with >90% removal efficiency.

## Figures and Tables

**Table 1:** FFD matrix of actual variable together with experimentally obtained analytical response (%RE).

Standard Run	pH	MA	ET	%RE
**1**	2	50	5	98.8
**2**	8	50	5	32.4
**3**	2	200	5	98.9
**4**	8	200	5	47.8
**5**	2	50	40	99.4
**6**	8	50	40	52.5
**7**	2	200	40	98.5
**8**	8	200	40	51.3
**9 (C)**	5	125	22.5	68.7
**10 (C)**	5	125	22.5	68.4
**11 (C)**	5	125	22.5	68.4
**12 (C)**	5	125	22.5	69.6
**13 (C)**	5	125	22.5	68.9
**14 (C)**	5	125	22.5	67.6
**15 (C)**	5	125	22.5	68.7
**16 (C)**	5	125	22.5	67.2

**Table 2:** Central composite design matrix and %removal efficiency as an analytical response.

Standard Run	pH	ET (min)	%RE
**1**	2.0	5.0	99.1
**2**	2.0	40.0	99.3
**3**	8.0	5.0	44.4
**4**	8.0	40.0	52.4
**5**	0.8	22.5	57.3
**6**	9.2	22.5	46.8
**7**	5.0	−2.2	7.1
**8**	5.0	47.2	98.6
**9 (C)**	5.0	22.5	91.2
**10 (C)**	5.0	22.5	93.0
**11 (C)**	5.0	22.5	89.1
**12 (C)**	5.0	22.5	90.4
**13 (C)**	5.0	22.5	89.4
**14 (C)**	5.0	22.5	92.9
**15 (C)**	5.0	22.5	90.7
**16 (C)**	5.0	22.5	88.3
**17 (C)**	5.0	22.5	90.2
**18 (C)**	5.0	22.5	90.0

**Table 3:** Isotherm parameters for adsorption of arsenic on MWCNT-Fe_3_O_4_@Zeo nanocomposite.

Isotherms	Parameters		R^2^
**Langmuir**	q_max_ (mg/g)	27.8	0.9922
	K_L_ (L μg^−1^)	4.0	
	R_L_	0.021–0.26	
**Freundlich**	K_F_ (L g^−1^)	0.42	0.9706
	n	3	
**Temkin**	K_T_ (L g^−1^)	0.018	0.9
	b_T_ (kJ mol^−1^)	833	
	B	2.974	
**Redlich-Peterson**	K_RP_ (L g^−1^)	1150	0.9909
	β	0.98	
	α	81.9	
**Sips**	K_S_ (L g^−1^)	0.93	0.9907
	n	1.05	
	q_max_ (mg g^−1)^	28.2	
**Hill**	q_H_ (mg g^−1)^	29.3	0.9912
	K_D_	0.78	
	n_H_	0.86	

**Table 4:** Comparison of removal efficiency of arsenic between different adsorbents.

	Adsorbent	Adsorption Capacity (mg/g)	pH	Refs
**As (III) and As (V)**	CeO_2_/Fe_2_O_3_/graphene nanocomposite	84–101	7.8	[29]
**As**	nano-TiO_2_/feldspar-embedded chitosan beads	3–6	4–10	[44]
**As(III) and As(V)**	zeolitic imidazolate framework-8 (ZIF-8)	50–60	7	[46]
**As(V)**	Moroccan clays	0.56–1.1	7	[45]
**As(V) and As(III)**	iron–zirconium (Fe–Zr) binary oxide	46 and 120	7	[47]
**As(V)**	amino functionalized glycidylmethacrylate-grafted-titanium dioxide densified cellulose	109	6	[48]
**As**	MWCNT-Fe_3_O_4_@Zeo	28	2.9	Current Study

**Table 5:** Concentration of As in real samples, n = 6

Samples	Initial (mg L^−1^)	After Adsorption (µg L^−1^)	%RE
**AMD 1**	7.02 ± 0.12	13.9 ± 0. 9	99.8 ± 1.2
**AMD 2**	5.24 ± 0.22	9.60 ± 0.31	99.8 ± 0.9
**ADM 3**	14.5 ± 0.9	957 ± 5	93.4 ± 1.3
**AMD 4**	7.52 ± 0.21	91.2 ± 1.1	98.7 ± 1.4
